# Advances in ferroptosis mechanisms and therapeutic potential in head and neck squamous cell carcinoma

**DOI:** 10.3389/fcell.2026.1885597

**Published:** 2026-08-28

**Authors:** Lijun Tang, Ling Lin, Chunfang Xu, Hua Guo

**Affiliations:** 1 Department of Nursing, Hunan Provincial People’s Hospital (The First Affiliated Hospital of Hunan Normal University), Changsha, Hunan, China; 2 Department of General Surgery, Xiangya Hospital, Central South University, Changsha, Hunan, China; 3 Department of Gastroenterology, Turpan People’s Hospital, Turpan, Xinjiang, China; 4 Department of Nursing, Turpan People’s Hospital, Turpan, Xinjiang, China

**Keywords:** chemoresistance, ferroptosis, head and neck squamous cell carcinoma, iron metabolism, Nrf2-ARE signaling, SLC7A11, GPX4

## Abstract

Head and neck squamous cell carcinoma (HNSCC) is a biologically aggressive malignancy characterized by late diagnosis, frequent recurrence, and poor response to conventional therapies. Cisplatin-based chemoradiotherapy remains the standard of care, yet widespread chemoresistance limits head and neck squamous cell carcinoma (HNSCC) clinical efficacy. Ferroptosis, an iron-dependent form of regulated cell death driven by lipid peroxidation, has emerged as a promising mechanism to overcome drug resistance. Recent studies elucidate multiple ferroptosis-related pathways in HNSCC, including SLC7A11-mediated cystine/glutamate transport, GPX4 and glutathione metabolism, Nrf2-ARE antioxidant signaling, iron and sulfur metabolism, HDAC and EMT modulation, and TP53 mutations. Pharmacologic inducers such as erastin, RSL3, sulfasalazine, dihydroartemisinin, and HDAC inhibitors effectively trigger ferroptosis and suppress tumor growth, both *in vitro* and *in vivo*, while combination strategies with photodynamic therapy or mutant TP53 modulation further enhance antitumor effects. Emerging predictive models incorporating ferroptosis-related genes, miRNAs, and lncRNAs enable prognostic stratification and may guide individualized therapy. This review highlights ferroptosis as a therapeutic vulnerability in HNSCC, offering new avenues to overcome chemoresistance and improve patient outcomes.

## 1 Introduction

Head and neck squamous cell carcinoma (HNSCC) is a highly aggressive and biologically heterogeneous malignancy frequently diagnosed at advanced stages, leading to high rates of local recurrence, distant metastasis, and mortality (Hartl et al., 2023; Wang H. et al., 2023). Although cisplatin-based chemoradiotherapy remains the standard of care, the inevitable development of acquired chemoresistance severely limits clinical efficacy and compromises long-term patient survival (De Felice et al., 2021; de Bakker et al., 2024). Furthermore, traditional dose escalation often leads to unacceptable systemic toxicities. Consequently, there is an urgent clinical need to identify novel therapeutic vulnerabilities and innovative, targeted strategies to overcome this refractory resistance and significantly improve clinical outcomes in HNSCC management (Tahara et al., 2025).

Ferroptosis, an iron-dependent form of regulated cell death driven by lethal lipid peroxidation, has recently emerged as a promising mechanism to circumvent tumor chemoresistance (Wang Y. et al., 2023; Xu et al., 2024; Tkachenko, 2025). Governed by distinct metabolic pathways—including the SLC7A11/GSH/GPX4 axis, iron metabolism, and Nrf2-ARE antioxidant signaling—ferroptosis offers a unique approach to eliminate resistant cancer cells (Zhou et al., 2024). Pharmacological induction of ferroptosis has demonstrated remarkable potential in suppressing HNSCC tumor growth and resensitizing cells to conventional therapies (Yang and Gu, 2024; Lee and Roh, 2026). This review comprehensively summarizes recent advances in the molecular mechanisms of ferroptosis in HNSCC, highlighting emerging therapeutic interventions, predictive biomarkers, and the critical crosstalk between ferroptosis and the tumor immune microenvironment, ultimately providing new avenues for individualized and effective HNSCC treatment.

## 2 Mechanisms of ferroptosis in cancer cell death

### 2.1 System Xc^−^/GSH/GPX4 axis

System Xc^−^ is a plasma membrane cystine–glutamate antiporter composed of the SLC7A11 light chain and the SLC3A2 heavy chain, which mediates the uptake of extracellular cystine in exchange for intracellular glutamate (Yang et al., 2023; Jyotsana et al., 2022). Imported cystine is rapidly reduced to cysteine, the rate-limiting substrate for glutathione (GSH) synthesis. GSH subsequently serves as an essential cofactor for GPX4, which converts PLOOHs into PLOHs (Yang and Gu, 2024). Consequently, Accordingly, inhibition of system Xc^−^ restricts cystine uptake, depletes intracellular GSH, compromises GPX4 activity, and promotes lethal lipid peroxidation (Koppula et al., 2021). Pharmacological inhibitors like sulfasalazine suppress system Xc^−^ activity, inducing cystine starvation, GSH depletion, and subsequent ferroptosis (Chen et al., 2021a). Similarly, RSL3 directly inactivates GPX4 by covalently interacting with its catalytic selenocysteine residue, thereby blocking lipid peroxide detoxification (Koppula et al., 2021; Peng et al., 2022) and triggering lethal lipid peroxidation (Wu et al., 2025; Kalishwaralal et al., 2025). Crucially, this metabolic axis intersects dynamically with antitumor immunity and inflammatory regulation. Activated CD8^+^ T cells secrete IFN-γ, which downregulates SLC3A2 and SLC7A11 expression via the JAK/STAT1 pathway, thereby restricting cystine uptake and sensitizing tumor cells to ferroptosis (Zhou et al., 2024; Liang et al., 2025). This mechanism establishes a direct biological link between immune checkpoint blockade and ferroptotic cell death.

### 2.2 Iron metabolism and ferritinophagy

Cellular iron homeostasis is tightly maintained through the coordinated regulation of iron uptake, intracellular trafficking, storage, utilization, recycling, and export (Ru et al., 2024). In the circulation, ferric iron (Fe^3+^) binds to transferrin (Tf), and the resulting Tf–Fe^3+^ complex is internalized into cells through transferrin receptor 1 (TfR1, encoded by TFRC)-mediated endocytosis (Zhang Y-Y. et al., 2024). Following endosomal acidification, Fe^3+^ is released from Tf and reduced to ferrous iron (Fe^2+^) by six-transmembrane epithelial antigen of prostate 3 (STEAP3), after which Fe^2+^ is transported into the cytosol via divalent metal transporter 1 (DMT1), thereby contributing to the intracellular labile iron pool that fuels ferroptosis (Li et al., 2025; Horváth et al., 2026; Akyol et al., 2025; Barra et al., 2024). The LIP supplies iron for essential biological processes, including mitochondrial respiration, DNA synthesis, and the activity of iron-dependent enzymes. However, excessive intracellular Fe^2+^ drives the Fenton reaction, producing highly reactive hydroxyl radicals that propagate phospholipid lipid peroxidation, compromise membrane integrity, and ultimately trigger ferroptosis (Zhou et al., 2024; Ru et al., 2024). To limit iron toxicity, surplus intracellular iron is sequestered within ferritin, which consists of ferritin heavy and light chains. Under conditions of iron demand or cellular stress, NCOA4 selectively binds ferritin and delivers it to lysosomes for degradation through ferritinophagy, thereby releasing stored Fe^2+^ and expanding the LIP (Huang et al., 2026; Zhao et al., 2025). Conversely, ferroportin (Fpn) exports excess intracellular iron and reduces ferroptosis susceptibility (Ru et al., 2024). Consequently, dysregulation of TFRC1, DMT1, ferritin, NCOA4, or Fpn profoundly alters cellular ferroptosis sensitivity.

Beyond tumor-intrinsic metabolism, iron homeostasis is dynamically shaped by inflammatory signaling within the tumor microenvironment (Zhang Y-Y. et al., 2024; Liang and Ferrara, 2020). Pro-inflammatory cytokines, including TNF-α, IL-1β, and IL-6, remodel iron handling by modulating ferritin expression, hepcidin-mediated iron retention, and macrophage iron recycling (Chibanda et al., 2023; Liang and Ferrara, 2020; DeRosa and Leftin, 2021). In tumor-associated macrophages, inflammatory activation can exacerbate iron sequestration and oxidative stress (Zhang Y-Y. et al., 2024; Schwan et al., 2024). Conversely, M2-like macrophages may foster tumor progression through immunosuppressive cytokines and dysregulated iron metabolism (Sun et al., 2021; Yang et al., 2025). Furthermore, TNF-α or IL-1β-induced NF-κB signaling can amplify inflammatory ROS production, synergizing with ferroptosis-related oxidative damage (Chen Y. et al., 2023; He et al., 2024). Collectively, these mechanisms underscore that iron-dependent ferroptosis is not merely a cell-autonomous metabolic process, but a highly immune-regulated event governed by macrophage dynamics, cytokine networks, and inflammatory signaling cascades.

### 2.3 Lipid peroxidation and membrane vulnerability

Lipid peroxidation is the central execution process of ferroptosis and primarily targets polyunsaturated fatty acid-containing phospholipids (PUFA-PLs) (Mortensen et al., 2023; Qiu et al., 2024). Owing to the presence of bis-allylic hydrogen atoms, PUFAs are particularly susceptible to hydrogen abstraction and free-radical propagation. Before incorporation into membrane phospholipids, free PUFAs are activated by ACSL4, which preferentially converts arachidonic acid and adrenic acid into their corresponding acyl-CoA derivatives (Ding et al., 2023; Kim JW. et al., 2023; Yang et al., 2024). These activated fatty acids are subsequently esterified into phosphatidylethanolamine by LPCAT3, generating ferroptosis-sensitive phospholipid substrates (Liu X. et al., 2024; Lin et al., 2026). Therefore, elevated ACSL4 and LPCAT3 activity increases membrane PUFA-PLs and enhances ferroptosis susceptibility, whereas enrichment of monounsaturated fatty acids through ACSL3-mediated lipid remodeling can reduce membrane vulnerability (Zhou et al., 2025; Koeken et al., 2026).

Oxidation of PUFA-containing phospholipids may occur enzymatically through lipoxygenases, particularly ALOX15, or nonenzymatically through iron-dependent radical reactions (Yi et al., 2025). The resulting phospholipid hydroperoxides initiate self-propagating oxidative chain reactions that disrupt membrane integrity. Ferroptosis-associated lipid peroxidation serves as a potent immunomodulatory signal (Wang and Lu, 2022; Wiernicki et al., 2022). During this process, oxidized phospholipids and reactive aldehydes, such as 4-HNE, act as danger-associated molecular patterns (DAMPs). Ferroptotic tumor cells release these DAMPs, including HMGB1, which activate pattern-recognition receptors on dendritic cells and macrophages (Deng et al., 2025; Nurkolis, 2026). While this cascade can acutely enhance antigen presentation and pro-inflammatory cytokine production, chronic or excessive lipid peroxidation paradoxically fosters immune exhaustion, persistent inflammation, and immunosuppressive remodeling of the tumor microenvironment (Wiernicki et al., 2022). Thus, the immunological consequences of ferroptosis are highly context-dependent, dictated by the timing, intensity, and cellular source of the lipid peroxidation.

### 2.4 Alternative antioxidant systems and ferroptosis-regulatory signaling pathways

Ferroptosis suppressor protein 1 (FSP1) constitutes a major GPX4-independent defense pathway. Following N-terminal myristoylation and localization to the plasma membrane, FSP1 functions as an NAD(P)H-dependent oxidoreductase that reduces coenzyme Q10 (CoQ10) to ubiquinol (Lee et al., 2026; Liu et al., 2025). Ubiquinol acts as a potent lipophilic radical-trapping antioxidant, interrupting lipid peroxidation chain reactions and thereby preserving membrane phospholipid integrity. Accordingly, increased FSP1 activity may confer resistance to GPX4 inhibition and sustain the survival of HNSCC cells under conditions of oxidative stress (Bersuker et al., 2019).

The GTP cyclohydrolase 1 (GCH1)/tetrahydrobiopterin (BH4) pathway represents another important GPX4-independent ferroptosis-suppressive system (Hu et al., 2022). GCH1 catalyzes the rate-limiting step in BH4 biosynthesis, whereas BH4 directly scavenges lipid-derived radicals and contributes to the maintenance of reduced CoQ10 pools (Kraft et al., 2020). Through these antioxidant activities, the GCH1/BH4 axis limits phospholipid hydroperoxide accumulation and reduces membrane susceptibility to ferroptotic injury (Hu et al., 2022; Chen et al., 2024). Functional cooperation between the FSP1/CoQ10 and GCH1/BH4 pathways may therefore provide a compensatory survival advantage when the GSH/GPX4 system is impaired. Beyond antioxidant detoxification, ferroptosis resistance is also mediated by the ESCRT-III, which facilitates the repair of damaged plasma membranes (Dai et al., 2020). Upon lipid peroxidation-induced membrane injury, ESCRT-III promotes membrane constriction, budding, and shedding of oxidized membrane regions (Pedrera et al., 2021). This membrane-repair response delays catastrophic membrane rupture and transiently preserves cellular viability during ferroptotic stress. These protective systems are further integrated by the Keap1/Nrf2 signaling pathway (Yu et al., 2025; Nakayama et al., 2025). Under basal conditions, Keap1 promotes the ubiquitination and proteasomal degradation of Nrf2. Oxidative or electrophilic stress disrupts Keap1-mediated repression, resulting in Nrf2 stabilization, nuclear translocation, and binding to antioxidant response elements (Li et al., 2020). Nrf2 subsequently induces a broad ferroptosis-protective transcriptional program involving SLC7A11, GPX4, ferritin, and ferroportin.

**FIGURE 1:**
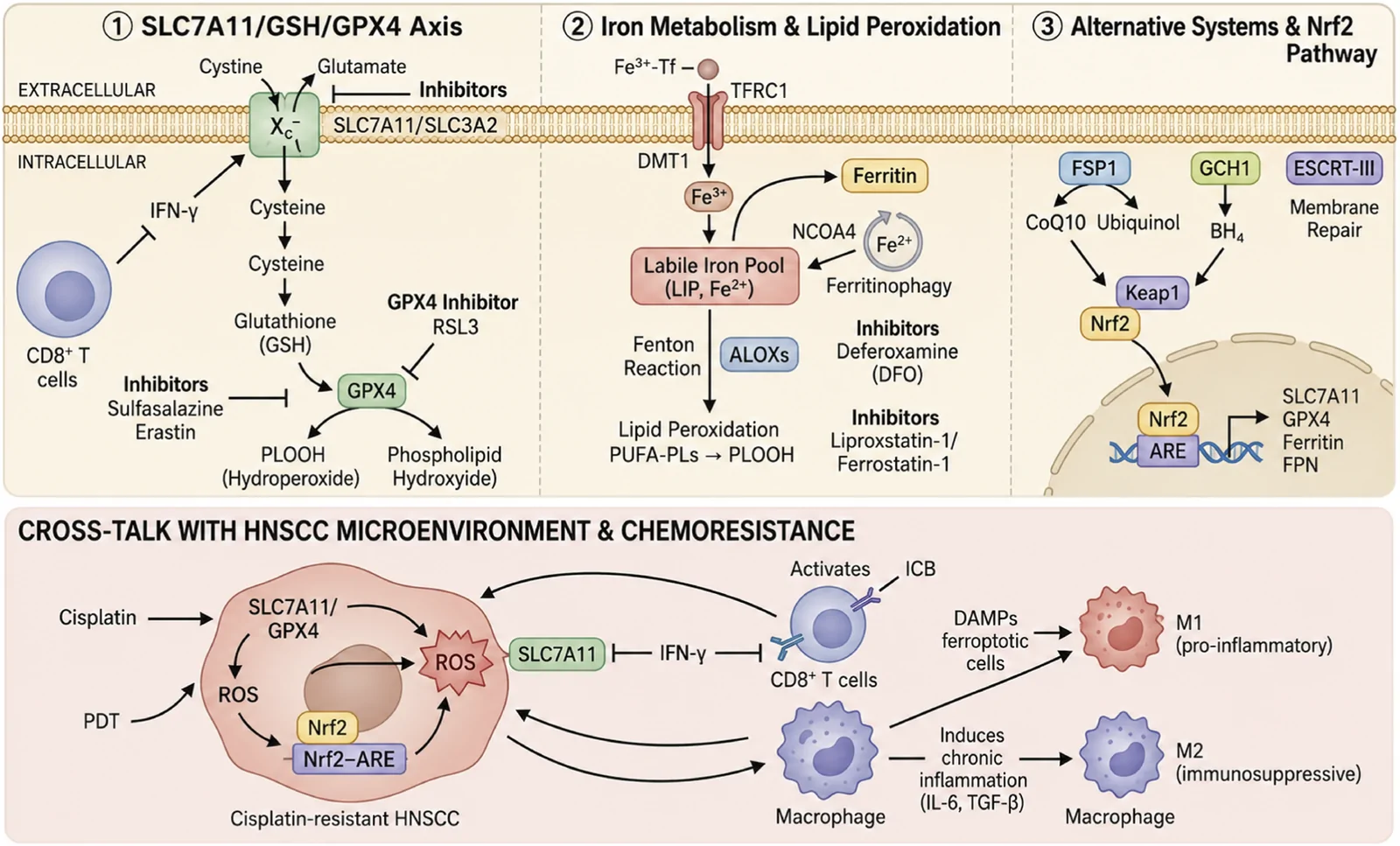
Key ferroptosis mechanisms in head and neck squamous cell carcinoma.

From an immunological perspective, ferroptosis is increasingly recognized as a bridge between tumor metabolism and antitumor immunity. CD8^+^ T-cell-derived IFN-γ can promote ferroptosis through JAK/STAT1-mediated suppression of system Xc^−^, whereas ferroptotic tumor cells may activate innate immune pathways through HMGB1 release, oxidized lipid mediators, and mitochondrial stress signals (Wang et al., 2019; Chen R. et al., 2023). The STING pathway may further amplify type I interferon-related inflammatory responses, enhancing dendritic-cell activation and T-cell priming (Yu et al., 2022; Ulrich-Lewis et al., 2022). Conversely, chronic inflammatory cytokines such as IL-6 and TNF-α can activate STAT3 and NF-κB signaling, which may promote tumor survival, immune evasion, and therapy resistance (Thuya et al., 2025; Mao et al., 2025). IL-10 and TGF-β can further establish an immunosuppressive microenvironment by promoting M2-like macrophage polarization and T-cell exhaustion (Sun et al., 2024; Xu et al., 2022) (Figure 1).

## 3 Advances in ferroptosis research in HNSCC

### 3.1 Regulation of ferroptosis by non-coding RNAs in HNSCC

Non-coding RNAs (ncRNAs), including miRNAs, lncRNAs, and circRNAs, have emerged as pivotal post-transcriptional regulators of ferroptosis in HNSCC. Several miRNAs, such as miR-7-5p and miR-527, modulate ferroptosis sensitivity by targeting the 3′ untranslated regions (3′UTRs) of key ferroptosis regulators like SLC7A11 and GPX4 (Tomita et al., 2021; Ma et al., 2023). For instance, miR-26a-5p has been shown to inhibit SLC7A11 expression by binding to its 3′UTR, thereby promoting erastin-induced ferroptosis in HNSCC cells and enhancing their sensitivity to ferroptotic cell death (Liang and Wu, 2023). This post-transcriptional repression of SLC7A11 disrupts antioxidant defenses, leading to lipid peroxidation and ferroptosis. Furthermore, coupling SLC7A11 inhibition with porphyrin derivatives amplifies intracellular ROS accumulation and ferroptotic cell death, successfully reversing cisplatin resistance in refractory HNSCC lines (Yang and Gu, 2024; Liu et al., 2023). By elevating intracellular ROS, modulating oxygen consumption, and downregulating SLC7A11, these nanoplatforms not only trigger potent ferroptotic cascades but also overcome the hypoxia-induced limitations inherent to conventional photodynamic therapy (PDT), thereby significantly enhancing overall PDT efficacy (Ma et al., 2026; Huang et al., 2023).

Beyond miRNAs, lncRNAs such as LINC00662 and LINC00511 function as competing endogenous RNAs (ceRNAs) by sponging ferroptosis-related miRNAs, thus relieving the suppression on ferroptosis core genes and facilitating tumor cells to evade ferroptotic death. For example, exosomal lncRNA FTX transferred from podoplanin-positive CAFs to HNSCC cells inhibits ferroptosis via the FTX/FEN1/ACSL4 axis, promoting tumor invasiveness (Li Y. et al., 2023). This highlights the complex intercellular communication mediated by lncRNAs in the tumor microenvironment that modulates ferroptosis resistance. Circular RNAs also contribute to ferroptosis regulation in HNSCC. In oral squamous cell carcinoma, circFNDC3B functions as a competing endogenous RNA for miR-520d-5p, thereby increasing SLC7A11 expression and suppressing ferroptosis. Silencing circFNDC3B reduces SLC7A11 and GPX4 expression, promotes ROS and Fe^2+^ accumulation, and enhances erastin-induced ferroptotic cell death (Yang et al., 2021). Emerging evidence indicates that circRNAs function as important regulators of ferroptosis in HNSCC-related malignancies by modulating ferroptosis-associated pathways, including the SLC7A11 and GPX4 axes (Yang et al., 2021; Zhao et al., 2026). This regulatory axis not only affects tumor cell survival but also impacts therapeutic responses, as ferroptosis induction has been linked to overcoming chemoresistance and enhancing radiosensitivity in HNSCC (Han et al., 2022; Nair et al., 2022). Therefore, targeting ncRNA-mediated ferroptosis pathways offers promising avenues for novel therapeutic strategies aimed at sensitizing HNSCC cells to ferroptosis and improving clinical outcomes.

### 3.2 Key signaling pathways interacting with ferroptosis in HNSCC

Ferroptosis in HNSCC is intricately regulated by several critical signaling pathways that modulate cellular redox balance, iron metabolism, and lipid peroxidation. The tumor suppressor p53 pathway plays a dual role in ferroptosis regulation by transcriptionally repressing SLC7A11 and activating pro-ferroptotic genes such as SAT1 and GLS2, thereby enhancing ferroptosis sensitivity under DNA damage stress (Fan et al., 2021). This p53-mediated modulation disrupts cystine uptake and glutathione synthesis, promoting lipid peroxidation and ferroptotic cell death (Mao et al., 2024). Conversely, the Nrf2/ARE pathway acts as a central driver of ferroptosis resistance in HNSCC by upregulating antioxidant genes including HO-1, NQO1, and FTH1, which collectively scavenge ROS and sequester iron ions to mitigate lipid peroxidation (Han et al., 2022; Ye et al., 2022). The activation of Nrf2 signaling confers protection against ferroptosis and is implicated in chemoresistance, as evidenced by the enhanced Nrf2/HO-1/xCT axis in cisplatin-resistant HNSCC cells, where its inhibition by agents like carnosic acid restores ferroptosis and drug sensitivity (Han et al., 2022). Additionally, aberrant activation of the PI3K/AKT/mTOR pathway, frequently observed in HNSCC, promotes lipid biosynthesis via upregulation of SREBP1, thereby suppressing ferroptosis and contributing to resistance against radiotherapy and platinum-based chemotherapy (Lu et al., 2022). In HNSCC, targeting the GPX4/GSH axis effectively triggers ferroptosis. For instance, chrysophanol reduces GPX4 levels and accumulates lipid peroxidation in FaDu and SAS oral squamous carcinoma cells (Zhao, 2025; Liu D. et al., 2024), while the direct GPX4 inhibitor RSL3 inactivates the enzyme to effectively induce ferroptotic death (Chen et al., 2025; Herrick et al., 2025). Artesunate (ART) elevates intracellular labile iron, depletes GSH, and accumulates ROS to exert significant ferroptosis-dependent antitumor effects (Liu and Li, 2026; Wang et al., 2024).

### 3.3 Iron and sulfur metabolism and the Nrf2-ARE pathway in HNSCC

RSL3 induces ferroptosis not only by inhibiting GPX4 but also via the PERK-ATF4-Sesn2/p62 axis to modulate the Nrf2-ARE pathway (Kim J-W. et al., 2023; Teng et al., 2023). Nrf2 interacts with Keap1 to enhance antioxidant response element (ARE) activity, reducing labile iron and conferring ferroptosis resistance (Tunc et al., 2025; Jiang et al., 2024). In HNSCC, Nrf2’s role is highly context-dependent. While transient activation protects normal epithelia from oxidative damage, sustained Nrf2 activation in malignant cells drives tumor progression (Tang et al., 2021; Jin et al., 2024). Persistent Keap1-Nrf2-ARE signaling transcriptionally upregulates cytoprotective genes, including SLC7A11, GPX4, and multidrug-resistance-associated transporters (Lee et al., 2025). These downstream effectors collectively increase cystine uptake, promote GSH biosynthesis, enhance GPX4-dependent detoxification of lipid hydroperoxides, regulate iron storage, and reduce intracellular ROS accumulation, thereby shielding tumor cells from ferroptosis and attenuating cisplatin-induced cytotoxicity (Han et al., 2022; Xu et al., 2023). Beyond cell-intrinsic antioxidant functions, Nrf2 activation is intricately linked to inflammatory signaling within the HNSCC tumor microenvironment (Patel et al., 2025). Therapies like cisplatin and radiotherapy trigger oxidative stress and release pro-inflammatory cytokines (IL-6, TNF-α, TGF-β), activating NF-κB, STAT3, PI3K/AKT, and MAPK pathways (Korns et al., 2024; Li J. et al., 2023; Luo C-G. et al., 2024). These cascades stabilize Nrf2 directly or indirectly via p62-mediated Keap1 sequestration, establishing a pro-survival state characterized by increased antioxidant capacity, reduced lipid ROS accumulation, and impaired ferroptotic cell death (Teng et al., 2023; Lee et al., 2025). Moreover, IL-6/STAT3 signaling may enhance SLC7A11 and GSH-associated metabolic programs, while TNF-α signaling can cooperate with Nrf2-driven cytoprotective transcription to maintain redox homeostasis under therapeutic stress (Lee et al., 2025; Xu et al., 2023; Li M. et al., 2022). Additionally, TGF-β-driven EMT remodels lipid metabolism and enhances cellular plasticity, thereby promoting therapeutic resistance and dynamically altering tumor-cell susceptibility to ferroptosis (Huang et al., 2025; Soukupova et al., 2021).

## 4 Ferroptosis and the immune microenvironment in HNSCC

CD8^+^ T cells represent one of the most important immune-cell populations linking ferroptosis to antitumor immunity (Liao et al., 2022; Lin et al., 2023). In HNSCC, effective antitumor immunity depends on the infiltration and functional activation of cytotoxic T lymphocytes (Ruffin et al., 2023; Xiang et al., 2023). Upon activation of cytotoxic CD8^+^ T cells by immune checkpoint blockade or tumor antigen recognition promotes the release of effector molecules, including IFN-γ, granzyme B, and TNF-α. Among these mediators, IFN-γ may enhance tumor-cell susceptibility to ferroptosis by activating JAK–STAT1-dependent transcriptional programs and suppressing the system Xc^−^ components SLC7A11 and SLC3A2 (Kong et al., 2021). Reduced cystine uptake subsequently limits glutathione synthesis and compromises GPX4-mediated detoxification of lipid hydroperoxides, resulting in lipid reactive oxygen species accumulation and increased ferroptotic vulnerability (Li F-J. et al., 2022). In parallel, IFN-γ–STAT1–IRF1 signaling may promote ACSL4-dependent incorporation of polyunsaturated fatty acids into membrane phospholipids, further facilitating lipid peroxidation (Gan, 2022). This mechanism provides a biological rationale for combining ferroptosis-inducing agents with PD-1/PD-L1 blockade in HNSCC, as immune checkpoint inhibitors may enhance CD8^+^ T-cell-derived IFN-γ, while ferroptosis inducers simultaneously lower the antioxidant threshold of tumor cells.

Macrophages are another key component of ferroptosis–immune crosstalk (Luo X. et al., 2024). Tumor-associated macrophages in HNSCC often display an M2-like immunosuppressive phenotype characterized by increased IL-10, TGF-β, ARG1, CD206, and VEGF expression, which contributes to immune escape, angiogenesis, invasion, and resistance to therapy (Guo et al., 2023; Lin et al., 2024). Ferroptotic tumor cells can release lipid peroxidation products, oxidized phospholipids, HMGB1, oxidatively damaged DNA, and other DAMPs, which engage pattern-recognition receptors on macrophages and activate NF-κB, NLRP3 inflammasome-, and cGAS–STING-dependent inflammatory programs (Mu et al., 2024; Cui et al., 2024; Chen et al., 2021b). This inflammatory stimulation may promote M1-like macrophage activation, increasing TNF-α, IL-1β, IL-12, iNOS, CXCL9, and CXCL10, thereby supporting T-cell recruitment and antitumor immunity (Zhang Y. et al., 2024; Shen et al., 2025). However, prolonged exposure to ferroptosis-associated oxidative stress may instead reinforce chronic inflammation, enhance IL-6/STAT3 and HIF-1α signaling, and promote CCL2-mediated monocyte recruitment and M2-like macrophage polarization (Nurkolis, 2026; Chen et al., 2022). Therefore, ferroptosis may either convert HNSCC into a more inflamed and immunotherapy-sensitive tumor or, under chronic inflammatory conditions, contribute to immune suppression and tumor progression.

## 5 Conclusion

Ferroptosis has emerged as a promising therapeutic strategy for overcoming treatment resistance in HNSCC. Increasing evidence demonstrates that ferroptosis is orchestrated through a complex regulatory network involving the SLC7A11/GSH/GPX4 axis, iron metabolism, lipid peroxidation, alternative antioxidant systems, and the Nrf2-ARE signaling pathway. These pathways not only determine the susceptibility of HNSCC cells to ferroptotic death but also interact extensively with inflammatory signaling and the tumor immune microenvironment. Recent studies further reveal that ferroptosis induction can enhance antitumor immunity by promoting CD8^+^ T-cell activity and modulating macrophage polarization, providing a strong biological rationale for combining ferroptosis-inducing agents with immune checkpoint inhibitors and conventional chemoradiotherapy.

Despite these advances, several challenges remain before ferroptosis-based therapies can be translated into routine clinical practice. First, no ferroptosis inducer has yet received regulatory approval for HNSCC, and the long-term safety, pharmacokinetics, and off-target toxicity of current compounds require comprehensive evaluation. Second, the substantial molecular heterogeneity of HNSCC, together with dynamic metabolic reprogramming and activation of compensatory antioxidant pathways such as Nrf2/FSP1/GCH1, may limit therapeutic efficacy and promote acquired resistance. Furthermore, reliable biomarkers capable of accurately predicting ferroptosis sensitivity and treatment response remain insufficiently validated in prospective clinical studies. Future investigations should integrate multi-omics technologies, single-cell and spatial transcriptomics, and functional validation to define ferroptosis-associated molecular subtypes and identify clinically actionable biomarkers. Rational combination strategies integrating ferroptosis induction with immunotherapy, targeted therapy, nanomedicine, or precision drug delivery are expected to maximize therapeutic efficacy while minimizing systemic toxicity. Collectively, continued mechanistic investigations and well-designed clinical studies will be essential for translating ferroptosis from an experimental concept into an effective precision therapeutic strategy for patients with HNSCC.
